# Subgroup analysis of the influence of body mass index on the association between serum lipids and cognitive function in Chinese population

**DOI:** 10.1186/s12944-020-01314-7

**Published:** 2020-06-08

**Authors:** Jiang Li, Yongtong Cao, Cheng Xiao

**Affiliations:** 1 Department of Laboratory Medicine, China-Japan Friendship Hospital, Beijing, 100029 China; 2 Institute of Clinical Medicine, China-Japan Friendship Hospital, Beijing, 100029 China; 3 Graduate School of Peking Union Medical College, Chinese Academy of Medical Sciences/Peking Union Medical College, Beijing, 100193 China

**Keywords:** Subgroup analysis, Body mass index, Cognitive function, Blood lipid profile, Chinese population, China health and nutrition survey

## Abstract

**Background:**

Previous studies reported that the association between lipid levels and cognitive function is related with gender, age and specific cognitive domains, but the influence of body mass index (BMI) on this association is limited. This triggered interest in exploring how serum lipids relate to cognitive function in different subgroups.

**Methods:**

Data was collected from 2009 wave and 2015 wave of China Health and Nutrition Survey (CHNS). Multivariable linear regression analyses examined serum lipids level as predictors of sex- and age-specific measure of cognitive function in different BMI levels, which were adjusted for nationality, BMI, systolic blood pressure (SBP), diastolic blood pressure (DBP), smoking status, alcohol consumption and education level.

**Results:**

Cognitive function score have different concentration curves in serum lipids quartile levels in different BMI categories. After adjustment for confounding factors, serum TG was positively associated with cognitive function score in underweight (β ± SE: 2.06 ± 0.88, *P* = 0.023) and obese (β ± SE: 1.44 ± 0.71, *P* = 0.045) male group, and serum HDL-C was positively associated with cognitive function score in overweight (β ± SE: 1.89 ± 0.92, *P* = 0.041) and obese (β ± SE: 5.04 ± 1.62, *P* = 0.002) female group. Serum TC was negatively associated with cognitive function score in overweight (β ± SE: − 2.55 ± 1.26, *P* = 0.043) mid-life adults, and serum HDL-C was positively associated with cognitive function score in overweight (β ± SE: 2.15 ± 0.94, *P* = 0.022) and obese (β ± SE: 5.33 ± 2.07, *P* = 0.011) older adults.

**Conclusion:**

The associations of serum lipids with cognitive function were related with BMI levels and differed between gender and age groups. This result indicated that better nutritional status has superior cognitive function performance.

## Introduction

Cognitive impairment is an obvious and important disorder among the elderly and affecting the life of the elderly, which is an intermediate stage between normal aging and dementia. It is especially important to test the physical and mental health and quality of life of elderly individuals [1]. This group of people represents a population at risk of developing dementia. In 2018, a systematic review showed that the pooled prevalence of mild cognitive impairment (MCI) in China was 14.71% and people of older age, of female sex, or living in rural areas or western China were associated with a higher prevalence of MCI [2]. In a study among of Chinese older adults aged 90 years and over [3], the prevalence of cognitive impairment increased to 57.8% overall and up to 67.2% among women. Other studies have shown similar results [4, 5] . These inconsistencies might be attributable to gender- and age-related differences in blood lipid profiles, aetiology of dementia and methods of cognitive assessment [2, 6].

Previous study speculated that associations of serum lipids with cognitive performance are inversely U-shaped or J-shaped [6]. These associations implied that participants with extremely low or high levels of serum lipids would be expected to show poorer cognitive performance. The possible pathophysiological mechanisms of high serum lipids with the risk of cognitive decline is that high level of cholesterol can contribute to an overproduction and accumulation of β-amyloid in the brain [7]. The possible explanation for low serum lipid levels with the risk of cognitive decline is the nutritional status of participants. Participants with malnutrition show alterations in the energetic profile, such as weight loss, reduced caloric, increased energy requirement, low lipid levels and cognitive function impairment [8, 9].

Two reviews concluded that high serum cholesterol associates with cognitive function and the association is strongly age-dependent [10, 11]. However the associations between serum or plasma triglyceride (TG) and cognitive function were complicated and associated with body mass index (BMI) and gender (Table 1). Yin et al. [12] found that high normal plasma TG was association with preservation of cognitive function while lower concentrations were not in the Chinese oldest-old. Two studies reported that lower TG was associated with higher cognitive scores in most cognitive domains [13] and better short-term memory [15]. Lv et al. [14] found that TG was associated with Mini Mental Status Examination (MMSE) score, but after adjustment for central obesity and other confounding factors, it was not associated with the risk of cognitive impairment. Parthasarathy et al. [16] even found that TG levels are inversely correlated with executive function in non-demented elderly adults.

**Table 1:** Reported association of serum/plasma triglycerides with cognitive function in previous studies

Source	Study/Population/study design	Cognitive function assessment methods	Regression models	Comparison and effect estimates (95% CI)	Adjustment for covariates	Main results
Yin et al. (2012) [12]	The Chinese Longitudinal Healthy Longevity Survey (CLHLS); 836 participants aged 80 and older; cross-sectional study	The Mini-Mental Status Examination (MMSE)	Model 1	0.67 (0.52–0.86)**	Age, sex, ethnicity and education	High normal plasma TG was association with preservation of cognitive function while lower concentrations were not in the Chinese oldest-old.
			Model 2	0.65 (0.50–0.84)**	Model 1 ± leisure activity, smoking, drinking and systolic blood pressure	
Janie et al. (2015) [13]	The Lothian Birth Cohort 1936 Study; 1043 participants; cross-sectional study and follow-up study	The Wechsler Adult Intelligence Scale-III (WAIS-III)	Model 1	Standardized regression coefficient: − 0.070*	Sex, age	Lower TG was associated with higher cognitive scores in most cognitive domains.
			Model 2	0.007	Model 1 ± age 11 IQ	
			Model 3	0.006	Model 2 ± occupational social class, statin use, and history of cardiovascular disease	
Lv et al. (2016) [14]	The Chinese Longitudinal Healthy Longevity Survey (CLHLS) in 2012; 2437 participants aged 65 and older; cross-sectional study	The Mini-Mental Status Examination (MMSE)	Model 1	Compared with the lowest tertile: 0.86 (0.74–1.00)*	Age, gender, marital status, residence and education level	TG was associated with MMSE score in linear regression models. But in multiple logistic regression model, TG levels were not associated with the risk of cognitive impairment.
			Model 2	0.88 (0.76–1.03)	Model 1 ± current cigarette smoking, current alcohol drinking, **central obesity**, sleep quality, anemia, hypertension, type 2 diabetes mellitus and CKD	
Kanoski et al. (2011) [15]	The Multi-Ethnic Study of Atherosclerosis (MESA) study: 6814 participants; cross-sectional study and follow-up study	Cognitive Ablilities Screening Instrument (CASI) version 2, Digit Symbol Coding (DSC) and Digit Span (DS)	Model 1	Standardized regression coefficient: 0.026*(Digit Symbol Coding Test) and − 0.020(Forward Digit Span Test)	age, sex and race/ethnicity	Lower TG was associated with better short-term memory.
			Model 2	0.030*(Digit Symbol Coding Test) and − 0.037*(Forward Digit Span Test)	Model 1 ± education, smoking status, pack-years of smoking, current alcohol insurance, foreign born status, physical activity, use of lipid-lowing medication, **waist:hip ratio**, height, diabetes, hypertension, CRP level, fibrinogen level, interleukin-6 level, APOE genotype, hypertension and concentrations of other lipids	
Reitz et al. (2004) [8]	4316 Medicare recipients aged 65 and older; cross-sectional and prospective community-based cohort	Vascular Dementia	Model 1	Compared with the lowest quartile: 0.82 (0.54–1.26)	sex, age, education and race	TG was not related with the risk of Vascular Dementia.
			Model 2	0.95 (0.58–1.56)	Model 1 ± **BMI**, APOE genotype, diabetes, heart disease and hypertension	
Parthasarathy et al. (2014) [16]	251 participants; cross-sectional study	Standardized neuropsychological tests, including the executive functioning measure (EXEC), and the memory measure (MEM)	Model 1	Standardized regression coefficient:-13.20*	age, education, gender,	TG levels are inversely correlated with executive function in non-demented elderly adults.
			Model 2	−10.47*	Model 1 ± TC, LDL, APOE4 status, Clinical Dementia Rating scores (CDR) and white matter microstructure.	

**Bold value** means the significant association of serum/plasma TG with cognitive function in previous studies

*: *P* < 0.05;

Most of research only focused on the total population and did not considered the influence of BMI on these associations, leading to some ambiguity with respect to potential BMI differences in serum lipids and cognitive function. So these inconsistent results and potential relationships triggered interest in exploring the influence of BMI on the association between serum lipids and cognitive function in different subgroups based on a nationally representative sample of the Chinese population.

## Methods

### Setting

The China Health and Nutrition Survey (CHNS) started in 1989. It was intended to represent a range of economic and demographic variation in China. Data of participants of CHNS came from 12 provinces (from north to south, including Heilongjiang, Liaoning, Shandong, Henan, Hubei, Hunan, Jiangsu, Guangxi, Guizhou, Ningxia, Shaanxi and Yunnan) and 3 municipal cities (Beijing, Shanghai and Chongqing). Blood biomarkers were tested for the first time in 2009. Details of the study design and sampling strategies are available at the World Wide Web site (https://www.cpc.unc.edu/projects/china) and elsewhere [17, 18].

All the documentation and procedures comply with Good Clinical Practice (GCP), Human Ethics Protocol Rules and related Chinese laws. The CHNS project was approved by the office of human research ethics of the University of North Carolina at Chapel Hill and the Human & Clinical Research Ethics Committee of China-Japan Friendship Hospital (Study ID: 07–1963). Approved consent forms and other documents are available online at the World Wide Web site (http://apps.research.unc.edu/irb/index.cfm?event=home.dashboard.irbStudyManagement&irb_id=07-1963).

### Data collection methods

The data of demographic, anthropometric, lifestyle, memory status and perceived stress index were collected by trained interviewers used a questionnaire. Height and weight were measured based on a standard protocol. Height was measured to the nearest 0.1 cm, and weight in lightweight clothing was measured to the nearest 0.1 kg. BMI was calculated as weight in kg divided by height in square metres.

Trained nurses drew fasting blood from participants’ antecubital vein in the morning. Blood samples were treatment (centrifuged at 3000 g for 10 mins at room temperature and separated into 9 aliquots) within 2 h of collection in local hospitals. Aliquots were storage in − 80 degree freezers.

Serum TG (Lot number: 192AIF), total cholesterol (TC) (Lot number: 203AIG), high-density lipoprotein cholesterol (HDL-C) (Lot number: 548AIE) and low-density lipoprotein cholesterol (LDL-C) (Lot number: 362AIG) were detected using the enzymatic colorimetric method (Kyowa Medex Co., Ltd., Takatsuki-shi, Osaka, Japan). The calibrators and control serums were provided by the department of laboratory medicine of China-Japan Friendship Hospital and had the same lot number.

### Definition of body size phenotypes and BMI levels

Participants were classified as follows [19]:

- Underweight: BMI < 18.5 kg/m^2^;
- Normal weight: BMI 18.5–23.9 kg/m^2^;
- Overweight: BMI 24.0–27.9 kg/ m^2^;
- Obese: BMI of 28–31.9 kg/m^2^;
- Severely obese: BMI ≥32.0 kg/m^2^.

### Definition of age levels

Participants were classified as follows [20]:

- Mid-life adults: < 65 years;
- Older adults: ≥65 years.

### Assessment of memory status and cognitive function

The global cognitive score was calculated using composite scores of memory, counting back and subtraction scores. The cognitive screening items of questionnaire used in CHNS included a subset of items from the telephone interview for cognitive status-modified [21, 22]. The questionnaire included two questions for assessing self-reported memory status and four tests for testing memory performance. The first question asked about memory status: “How is your memory?” The response categories were “very good”, “good”, “OK”, “bad” and “very bad”. Those who reported “bad” or “very bad” were defined as having a poor memory. The second question asked about changes in memory status: “In the past twelve months, how has your memory changed?” The response categories were “improved”, “stayed the same” and “deteriorated”. Those who reported “deteriorated” were defined as self-reported memory decline.

The following four tests were related to cognitive function on specific memory tasks. Four tests were administered in the following order: 1) the first was a word list memory test for immediate memory, in which an examiner read a list of 10 unrelated words at 2-s intervals and immediately asked the participant to repeat to them as many words as possible in any order (score 10); 2) the following two tests were mind control ability tests in which an examiner counted backward from 20 to 1 (score 2) and calculated 100 minus 7 and subtracted 7 again and again (score 5); and 3) the last test was a test for delayed memory, in which a list of words were repeated to an examiner after a period of time (score 10). An orientation test was not included in the analysis as it was only assessed in 2015 wave. The cognitive function score was used to assess memory performance, which was the sum of the scores of the four tests and could range from 0 to 27 points. The Cronbach alpha internal consistency coefficient of this scale was 0.73, which is above the acceptable cut-off value of 0.70.

### Study population

**Fig. 1:**
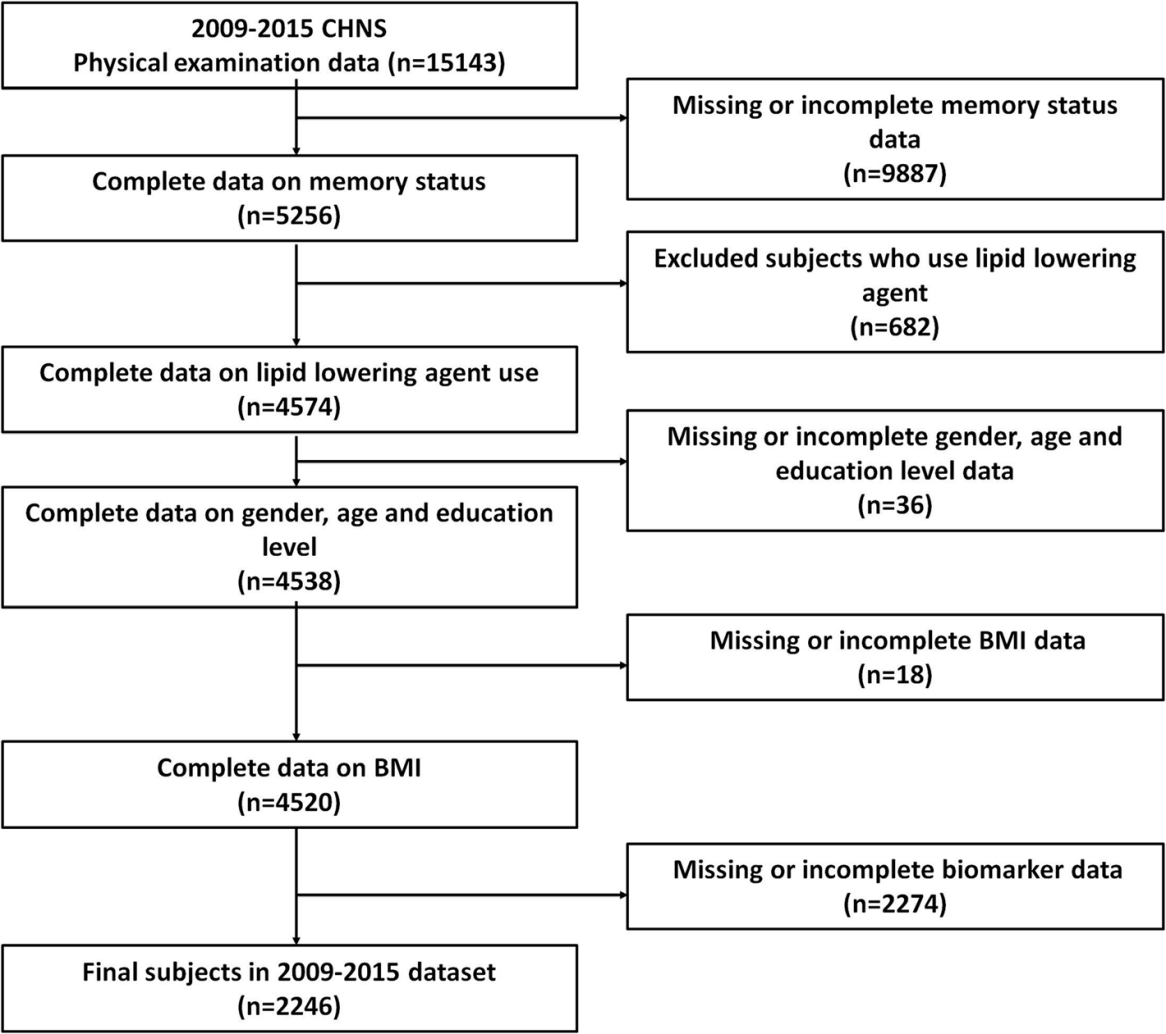
Flow chart of the participant selection

In total, 15,143 CHNS participants were included, and 5256 participants had complete memory status data. After excluding the participants with use lipid lowering agent, 4574 participants remained. After further excluding the participants with missing or incomplete gender, age and education level data, 4538 participants remained. Subsequently, 18 participants with missing BMI data and 2274 participants with missing biomarker data were excluded, 2246 participants (1120 men and 1126 women) remained (Fig. 1).

### Statistical methods

The current study was restricted to 2246 participants to examine the influence of BMI on the association between serum lipids and cognitive function among Chinese population. For the baseline characteristics of participants, all data are shown as the means±standard deviations (SDs) for normal variables and as medians (interquartile ranges) for skewed variables. Differences in characteristics between gender and age subgroups were tested for significance. The unpaired t-test or Mann-Whitney U test was used to compare the differences between continuous variables, and the chi-square test was used for categorical variables.

Multivariable linear regression analyses examined serum lipids level as predictors of gender- and age-specific measure of cognitive function in different BMI levels, which were adjusted for confounding factors, including gender, age, nationality, BMI, systolic blood pressure (SBP), diastolic blood pressure (DBP), smoking status, alcohol consumption and education level. The statistical analysis was performed with program R 3.4.3.

## Results

### Baseline characteristics of the study participants

The baseline characteristics of the participants were described separately for different gender subgroups (1120 men and 1126 women) and age subgroups (1160 mid-life adults and 1086 older adults) (Table 2). A total of 2246 participants, aged 65.64 ± 7.52 years (range, 55–94 years) with a BMI of 23.98 ± 3.54 kg/m^2^, were included in this study. The women showed higher BMI levels (24.58 ± 3.64 vs. 23.74 ± 3.45, *P* < 0.001), lower rates of current smoking (4.17% vs. 53.13%, *P* < 0.001) and alcohol consumption (4.88% vs. 50.54%, *P* < 0.001), lower education level and poorer self-reported memory status than the men. The women also showed higher serum lipids levels than the men, including TG [1.44(1.23) vs. 1.23(1.10), *P* < 0.001], TC [5.12(1.25) vs. 4.80(1.22), *P* < 0.001], HDL-C [1.43(0.48) vs. 1.38(0.49), *P* = 0.001] and LDL-C [3.21(1.21) vs. 2.96(1.14), *P* < 0.001]. The older adults showed higher SBP levels (139.45 ± 19.48 vs. 133.87 ± 18.38, *P* < 0.001), lower BMI levels (24.00 ± 3.64 vs. 24.35 ± 3.56, *P* = 0.013) and cognitive function score (12.34 ± 4.76 vs. 13.65 ± 4.63, *P* < 0.001), lower rates of alcohol consumption (23.66% vs. 31.38%, *P* < 0.001), lower education level and poorer self-reported memory status than mid-life adults. Other characteristics were not significantly different.

**Table 2:** Baseline characteristics of the study participants in different subgroups

Item	Subgroups					
	Sex groups			Age groups		
	Men	Women	*P* value	Mid-life adults	Older adults	*P* value
n	1120	1126		1160	1086	
Age, years	65.87 ± 7.71	65.29 ± 7.25	0.139	59.70 ± 2.78	71.87 ± 5.58	**< 0.001**
BMI, kg/m^2^	23.76 ± 3.48	24.61 ± 3.67	**< 0.001**	24.35 ± 3.56	24.00 ± 3.64	**0.013**
SBP, mmHg	136.45 ± 18.83	136.68 ± 19.42	0.964	133.87 ± 18.38	139.45 ± 19.48	**< 0.001**
DBP, mmHg	83.49 ± 10.91	82.36 ± 10.18	0.069	83.36 ± 10.69	82.46 ± 10.41	0.111
Nationality, Han, n(%)	992 (88.57)	1011 (89.79)	0.390	1053 (90.78)	950 (87.48)	**0.014**
Smokers, n(%)	595 (53.13)	47 (4.17)	**< 0.001**	339 (29.22)	303 (27.90)	0.518
Alcohol use/last year, n(%)	566 (50.54)	55 (4.88)	**< 0.001**	364 (31.38)	257 (23.66)	**< 0.001**
Education level, n(%)			**< 0.001**			**< 0.001**
Low	426 (38.04)	678 (60.21)		463 (39.91)	641 (59.02)	
Medium	565 (50.45)	352 (31.26)		597 (51.47)	320 (29.47)	
High	129 (11.52)	96 (8.53)		100 (8.62)	125 (11.51)	
Self-reported memory status, n(%)			**0.012**			**< 0.001**
Very good	97 (8.66)	67 (5.95)		117 (10.09)	47 (4.33)	
Good	293 (26.16)	260 (23.09)		339 (29.22)	214 (19.71)	
OK	520 (46.43)	544 (48.31)		549 (47.33)	515 (47.42)	
Bad	191 (17.05)	237 (21.05)		142 (12.24)	286 (26.34)	
Very bad	19 (1.70)	18 (1.60)		13 (1.12)	24 (2.21)	
Self-reported changes in memory status, n(%)			**0.011**			**< 0.001**
Improved or stayed the same	614 (54.82)	553 (49.11)		697 (60.09)	470 (43.28)	
Deteriorated	506 (45.18)	573 (50.89)		463 (39.91)	616 (56.72)	
Cognitive function score	13.20 ± 4.71	12.83 ± 4.76	0.061	13.65 ± 4.63	12.34 ± 4.76	**< 0.001**
Serum lipids						
TG, mmol/L	1.23 (1.10)	1.44 (1.23)	**< 0.001**	1.35 (1.23)	1.34 (1.12)	0.218
TC, mmol/L	4.80 (1.22)	5.12 (1.25)	**< 0.001**	4.96 (1.25)	4.99 (1.29)	0.911
HDL, mmol/L	1.38 (0.49)	1.43 (0.49)	**0.001**	1.42 (0.50)	1.40 (0.47)	0.652
LDL, mmol/L	2.96 (1.14)	3.21 (1.22)	**< 0.001**	3.06 (1.19)	3.09 (1.19)	0.173

Values are presented as the mean ± SD, median (interquartile range) or number (percent)

Bold value means *P* < 0.05

*BMI* body mass index, *SBP* systolic blood pressure, *DBP* diastolic blood pressure, *TG* triglycerides, *TC* total cholesterol, *HDL-C* high-density lipoprotein cholesterol, *LDL-C* low-density lipoprotein cholesterol

### Distribution of cognitive function in quartile levels of serum lipids in different BMI categories

**Fig. 2:**
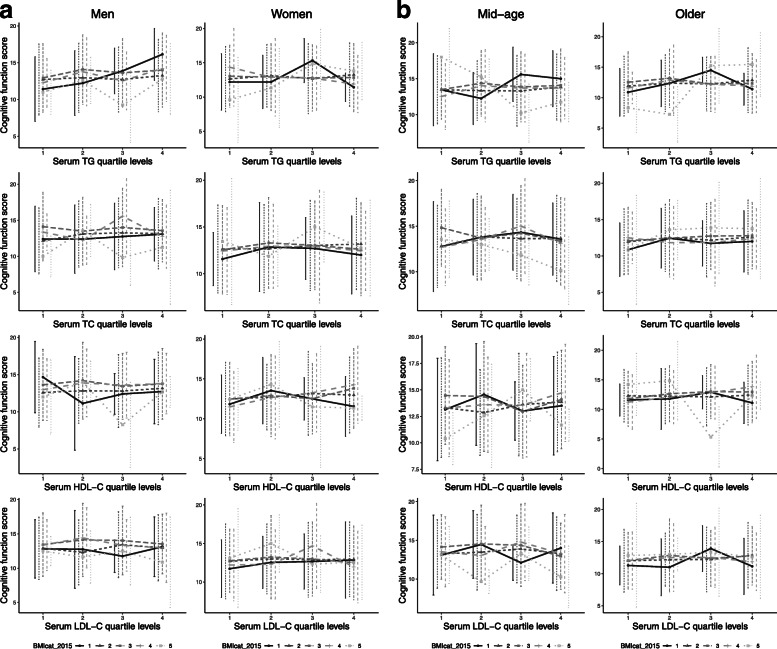
**a** Distribution of cognitive function in quartile levels of serum lipids in different BMI categories in gender subgroups. **b** Distribution of cognitive function in quartile levels of serum lipids in different BMI categories in age subgroups

Gender- and age-specific distribution of cognitive function in different BMI categories were shown in Fig. 2a and b. Participants were divided into four groups according to quartile levels of serum lipids. Cognitive function scores have different curves in different BMI categories.

### Subgroup analysis of the influence of BMI on the association between serum lipids and cognitive function

Multivariable linear regression analyses were performed separately to examine gender- and age-specific associations between serum lipids and cognitive function in different BMI categories (Table 3). After adjustment for confounding factors, serum TG was positively associated with cognitive function score in underweight (β ± SE: 2.06 ± 0.88, *P* = 0.023) and obese (β ± SE: 1.44 ± 0.71, *P* = 0.045) male group, and serum HDL-C was positively associated with cognitive function score in overweight (β ± SE: 1.89 ± 0.92, *P* = 0.041) and obese (β ± SE: 5.04 ± 1.62, *P* = 0.002) female group. Serum TC was negatively associated with cognitive function score in overweight (β ± SE: − 2.55 ± 1.26, *P* = 0.043) mid-life adults, and serum HDL-C was positively associated with cognitive function score in overweight (β ± SE: 2.15 ± 0.94, *P* = 0.022) and obese (β ± SE: 5.33 ± 2.07, *P* = 0.011) older adults.

**Table 3:** Sex-specific association of serum lipids with cognitive function score in different BMI categories (Multivariable linear regression)

Parameter	Status	Cognitive function score^a^															
		Men				Women				Mid-life adults				Older adults			
		β	SE	t	*P* value	β	SE	t	*P* value	β	SE	t	*P* value	β	SE	t	*P* value
**TG, mmol/L**	**Underweight**	**2.06**	**0.88**	**2.35**	**0.023**	−0.86	1.13	−0.76	0.452	0.25	0.81	0.31	0.758	− 0.13	1.02	− 0.13	0.898
	**Normal weight**	0.26	0.33	0.79	0.429	0.18	0.38	0.48	0.629	0.16	0.32	0.50	0.621	0.30	0.38	0.80	0.426
	**Overweight**	−0.12	0.40	−0.29	0.773	− 0.12	0.41	− 0.29	0.775	0.16	0.38	0.43	0.671	−0.43	− 0.43	− 0.99	0.323
	**Obese**	**1.44**	**0.71**	**2.03**	**0.045**	−1.05	0.79	− 1.34	0.184	0.29	0.67	0.43	0.669	−0.36	0.93	−0.39	0.700
	**Severely obese**	1.92	1.90	1.01	0.339	1.63	1.67	0.97	0.344	−2.71	1.56	−1.74	0.102	4.05	2.47	1.64	0.127
**TC, mmol/L**	**Underweight**	3.67	2.91	1.26	0.210	2.18	3.53	0.62	0.540	4.84	2.73	1.78	0.085	−1.64	3.09	−0.53	0.598
	**Normal weight**	1.62	1.09	1.49	0.136	0.42	1.12	0.37	0.712	1.11	1.05	1.06	0.288	0.92	1.16	0.80	0.425
	**Overweight**	−1.66	1.28	−1.29	0.198	−0.39	1.34	−0.29	0.770	**−2.55**	**1.26**	**−2.03**	**0.043**	0.56	1.33	0.43	0.671
	**Obese**	−0.28	2.26	−0.12	0.903	2.32	2.08	1.11	0.267	2.27	2.16	1.05	0.295	0.01	2.22	0.00	0.996
	**Severely obese**	−4.84	11.37	−0.43	0.680	1.04	5.11	0.20	0.841	−3.23	5.05	−0.64	0.532	−1.71	13.70	−0.13	0.903
**HDL, mmol/L**	**Underweight**	1.79	1.93	0.93	0.358	−1.89	2.53	−0.75	0.460	1.43	2.03	0.71	0.486	−0.67	2.28	−0.29	0.772
	**Normal weight**	0.13	0.70	0.19	0.849	0.79	0.88	0.91	0.365	0.34	0.73	0.47	0.642	0.56	0.78	0.72	0.474
	**Overweight**	0.60	0.90	0.66	0.508	**1.89**	**0.92**	**2.05**	**0.041**	0.66	0.86	0.77	0.442	**2.15**	**0.94**	**2.30**	**0.022**
	**Obese**	1.37	1.81	0.76	0.451	**5.04**	**1.62**	**3.10**	**0.002**	2.39	1.55	1.54	0.126	**5.33**	**2.07**	**2.58**	**0.011**
	**Severely obese**	−0.94	4.37	−0.22	0.835	2.17	4.72	0.46	0.650	6.53	3.53	1.85	0.083	−2.55	5.81	−0.44	0.669
**LDL, mmol/L**	**Underweight**	−1.10	1.64	−0.67	0.505	2.90	2.14	1.35	0.184	2.27	1.58	1.44	0.159	−1.29	1.85	−0.70	0.487
	**Normal weight**	0.77	0.61	1.27	0.205	−0.12	0.70	−0.17	0.869	0.18	0.61	0.30	0.763	0.55	0.69	0.79	0.428
	**Overweight**	−0.33	0.67	− 0.50	0.620	− 0.54	0.80	− 0.68	0.498	− 0.96	0.70	−1.36	0.173	−0.11	0.74	−0.15	0.879
	**Obese**	−1.64	1.27	−1.29	0.200	1.13	1.29	0.88	0.383	0.54	1.24	0.43	0.667	−0.46	1.36	−0.34	0.734
	**Severely obese**	−8.23	5.62	−1.47	0.177	−2.53	2.00	−1.27	0.221	−0.97	2.76	−0.35	0.731	−4.20	3.53	−1.19	0.257

Regression model (age subgroups): adjusted for gender, age, nationality, BMI, SBP, DBP, smoking status, alcohol consumption and education level;

Bold value means *P* < 0.05

*β* Unstandardized Beta, *SE* standard Error, *TG* triglycerides, *TC* total cholesterol, *HDL-C* high-density lipoprotein cholesterol, *LDL-C* low-density lipoprotein cholesterol

^**a**^ Regression model (gender subgroups): adjusted for age, nationality, BMI, SBP, DBP, smoking status, alcohol consumption and education level

## Discussion

This study investigated the influence of BMI on the association between serum lipids and cognitive function in different subgroups based on a nationally representative sample of the Chinese elderly population for the first time. A total of 2246 participants (1120 men and 1126 women), aged 65.64 ± 7.52 years (range, 55–94 years) with a BMI of 23.98 ± 3.54 kg/m^2^, were included in this study. Some of the results of this study were consistent with previous research results, and with general cognition. Serum TC was negatively associated with cognitive function score in overweight mid-life adults, and serum HDL-C was positively associated with cognitive function score in overweight and obese female group and older adults. But some of the results were inconsistent with general cognition. Serum TG was positively associated with cognitive function score in underweight and obese male group. It confirmed that serum lipids were related with cognitive function differed between gender- and age-subgroups in different BMI categories, and indicated that nutritional status was related with cognitive function performance.

Accumulating evidence suggests that lipid levels are associated with cognitive function and dementia, but results of previous studies showed that the association between lipid levels and cognitive function might be complicated and related with gender [6, 11], age [13] and specific cognitive domains [3]. Lu et al. [6] speculated that associations of serum lipids with cognitive performance is inversely U-shaped or J-shaped. Solomon et al. [23] reported a bidirectional relationship between TC and poor cognitive status and observed a tendency to an interaction between sex and TC changes over time in relation to late-life cognition, but due to size limitation, they could not draw definite conclusions. Ancelin et al. [24] reported that hypercholesterolemic late-life pattern (high TC, low HDL-C, high LDL-C) was related to an increased risk of cognitive function impairment in elderly men. But they also observed an unexpected association with low TC and LDL-C levels at the same time. The possible bidirectional pathophysiological mechanisms of serum lipids with the risk of cognitive decline is that high level of cholesterol can contribute to an overproduction and accumulation of β-amyloid in the brain [7] and cholesterol is an indispensible component of neuronal and glial membranes [25]. Participants with malnutrition show alterations in the energetic profile as weight loss, reduced caloric, increased energy requirement, low lipid levels and cognitive function impairment [8, 9].

This study showed that gender-specific associations between serum lipids and cognitive function. Serum TG was positively associated with cognitive function score in underweight and obese male group and serum HDL-C was positively associated with cognitive function score in overweight and obese female group. Two prospective studies on metabolic syndrome have examined cognitive decline in women specifically, finding no significant association between low HDL-C or high TG and decline on global cognitive performance [26, 27]. These gender-specific inconsistent associations may be related with genetic vulnerability, serum lipid patterns, hormonal factors and the differences of cognitive function between male and female brains [24, 28, 29]. Gender-specific differences in lipid metabolism are the consequence of the action of sex chromosomes and sex-specific hormones. Women store more lipids and have higher percent body-fat, less visceral white adipose tissue and more subcutaneous adipose tissue than men. Women have the higher rate of TG synthesis compared to men [30]. Gender-specific effects in lipid metabolism remain to be further elucidated.

Serum lipids are considered to be biomarkers of malnutrition [31] and serum TG may be a meaningful indicator of nutrition status [12, 32]. TGs can increase the blood-brain barrier transport of insulin, which can improve cognition function [33]. And higher serum TG indicates an abundance of circulating fatty acids, which can protect cognitive function and decrease the risk of dementia. This result is similar to some previous studies. Yin et al. [12] found that high normal plasma TG was associated with preservation of cognitive function in Chinese oldest-old and Ancelin et al. [24] found that TG levels were associated with a decreased risk of Alzheimer’s disease in women. The reasons for these relations remain to be clarified.

Studies on the influence of body mass index on the association between serum lipids and cognitive function are limited. There is no exploring the directly relationship between serum lipids and cognitive function in different BMI levels. Lv et al. [14] found that TG was associated with MMSE score, but after adjustment for central obesity and other confounding factors, it was not associated with the risk of cognitive impairment. Of the similar studies which have examined whole samples without BMI stratification, some cross-sectional and prospective studies found inconsistent associations with serum TG (Table 1). The role of serum lipids in different BMI levels in the elderly population remains unclear. Duo to the fact that there are relatively insufficient research about the dyslipidemia and cognitive function with BMI stratification and previous findings have suggested that the impact of extremely low BMI on cognitive function is significant [34], the present study may be of special importance in filling this gap.

### Study strengths and limitations

The strength of this study is related to large sample size of Chinese population to focus on the influence of BMI on the association between serum lipids and cognitive function in different sex and age subgroups.

There were several limitations of this study. First, the number and type of cognitive function tests performed at this study were limited and were not likely to provide particularly precise individual cognitive function score, such as an orientation test was not included in the analysis as it was only assessed in 2015 wave. Second, there was only 1 time point of measurement of serum lipids and assessment of cognitive function. Longitudinal analysis of the temporal relationship between serum lipids levels and cognitive function was not possible. Third, the data was adjusted by multiple confounding factors but cannot exclude the possibility of residual bias due to unmeasured confounders. Fourth, because it was an observational study, results could have been confounded by indication bias and cannot show direct causality of BMI and serum lipids with cognitive function. Last, although participants who use lipid-lowering agent were excluded, participants with hormone treatment were not excluded.

## Conclusions

Results of this study showed that the relationship between serum lipids and cognitive function was related with BMI levels and differed between subgroups. Serum TG was positively associated with cognitive function score in underweight and obese male group, and serum HDL-C was positively associated with cognitive function score in overweight and obese female group and older adults. These findings suggest that the levels of blood lipids are not as low as possible. Better nutritional status may be beneficial to cognitive function. Such analytical method needs to be further replicated and it could benefit from corresponding analysis.

## Data Availability

Details of the study design, sampling strategies and data are available at the World Wide Web site (https://www.cpc.unc.edu/projects/china).

## Abbreviations

BMI: Body mass index
CHNS: China Health and Nutrition Survey
TG: Triglyceride
TC: Total cholesterol
HDL-C: High-density lipoprotein cholesterol
LDL-C: Low-density lipoprotein cholesterol
SBP: Systolic blood pressure
DBP: Diastolic blood pressure
